# Association between triglyceride-glucose index and total trabecular bone score in US adults: A cross-sectional study

**DOI:** 10.1097/MD.0000000000051014

**Published:** 2026-10-23

**Authors:** Haichao Xu, Hao Xu, Jianyang Luo, Jianfeng Zhao

**Affiliations:** a Department of Orthopaedics, Shaoxing Second Hospital, Shaoxing, China.

**Keywords:** NHANES, osteoporosis, trabecular bone score, triglyceride-glucose index

## Abstract

This study examines the association between the triglyceride-glucose (TyG) index and trabecular bone score (TBS) among US adults. In this cross-sectional analysis of National Health and Nutrition Examination Survey 2005 to 2008, the descriptive cohort included 3880 adults with TBS and fasting laboratory data; 3571 with positive fasting-subsample weights contributed to survey-weighted regression. TyG was calculated as ln(triglycerides [mg/dL] × glucose [mg/dL]/2) and is dimensionless. Covariate missingness was handled by multiple imputation. The primary model excluded bone mineral density (BMD) to avoid overadjustment; BMD-adjusted, complete-case, and renal-function sensitivity models were fitted. In the primary model, higher TyG was associated with lower TBS (β per 1-unit increase = −0.0097; 95% confidence interval = −0.0182 to −0.0012; *P* = .026). Restricted cubic spline showed nonlinearity (*P*-overall < .001; *P*-nonlinear < .001). Notably, across TyG quartiles, spine BMD remained stable (*P* = .104), whereas TBS declined substantially (from 1.446 to 1.310, *P* < .001), suggesting TyG specifically associates with bone quality rather than quantity. An exploratory two-segment knot at TyG 8.58 requires validation and is not a clinical threshold. Higher TyG is modestly associated with lower TBS, independent of BMD. While causal inference is precluded by cross-sectional design, TyG – calculated from routine labs at no extra cost – may serve as a pragmatic signal to prompt TBS evaluation in metabolically unhealthy patients, particularly obese males, even when BMD is normal. Longitudinal validation is needed.

## 1. Introduction

With severe consequences for both physical and mental health, osteoporosis serves as a chronic systemic bone disease marked by reduced bone density and structural degradation that leaves bones brittle and more prone to fractures.^[1]^ Research has indicated an increasing osteoporosis incidence within middle-aged and older populations. In the USA, 10.2 million occurrences of the disease existed in those over 50 in 2010, and the expected case number in 2030 is expected to rise to 13.5 million.^[2,3]^ Osteoporosis presents serious economic and public health issues, as the world grapples with the escalating challenges of an aging population. Bone mineral density (BMD) measurement is a dependable way for osteoporosis diagnosis. Relying solely on BMD for assessing fracture risk may be insufficient, particularly for individuals with normal or high BMD values.^[4]^

A novel method called trabecular bone score (TBS) analyzes grayscale textural data from lumbar spine dual-energy X-ray absorptiometry (DXA) graphs to determine bone quality.^[5]^ TBS not only aids in detecting degradation in bone microarchitecture despite normal bone density but also offers improved predictive accuracy for osteoporotic fractures when compared to BMD.^[6,7]^ TBS is an indirect measure of bone quality, and studies have shown that TBS is an independent predictor of fracture risk.^[8]^ As a result, TBS, which assesses bone microstructure and quality, has garnered significant attention.

A new biomarker called the triglyceride-glucose (TyG) index uses easily accessible clinical data, specifically fasting triglyceride (TG) and glucose levels, which are frequently measured in clinical settings. Earlier research has underscored the potential connection between abnormalities in glucose and lipid metabolism and the initiation of osteoporosis. Notably, the TyG index has emerged as a surrogate marker, demonstrating utility in identifying individuals at risk for metabolic disorders.^[9–11]^ Numerous studies have examined the relationship between type 2 diabetes mellitus (T2DM) and BMD, with differing findings. Some researchers have found that people with T2DM have decreased BMD, while others have found normal or even greater BMD levels in these individuals.^[12,13]^ Furthermore, some research indicates that TBS is a more accurate measure of bone health in diabetics than BMD.^[14]^ However, there is still a lack of information in the literature about the correlation between TBS and glycolipid metabolism within the general population. To fully comprehend this association in the broader population, more research is necessary.

The minimum age requirement for research participants was 20 years old, and information from the National Health and Nutrition Examination Survey (NHANES) database was used. The principal study aim was the research on the connection between TBS and the TyG index.

## 2. Materials and methods

### 2.1. Data sources and population studies

NHANES uses a multistage, stratified probability design to represent the noninstitutionalized US population. Among 20,497 participants in the 2005 to 2008 cycles, 9583 were younger than 20 years, 393 were pregnant at examination, 2133 lacked a valid TBS value, and 4508 lacked fasting TG or glucose measurements, leaving a descriptive cohort of 3880. Of these, 309 had a zero fasting-subsample weight and therefore did not contribute to survey-weighted regression; the effective regression sample was 3571 (Fig. 1). Characteristics of included and excluded age-eligible, nonpregnant adults are reported in Table S1, Supplemental Digital Content 1.

**Figure 1.**
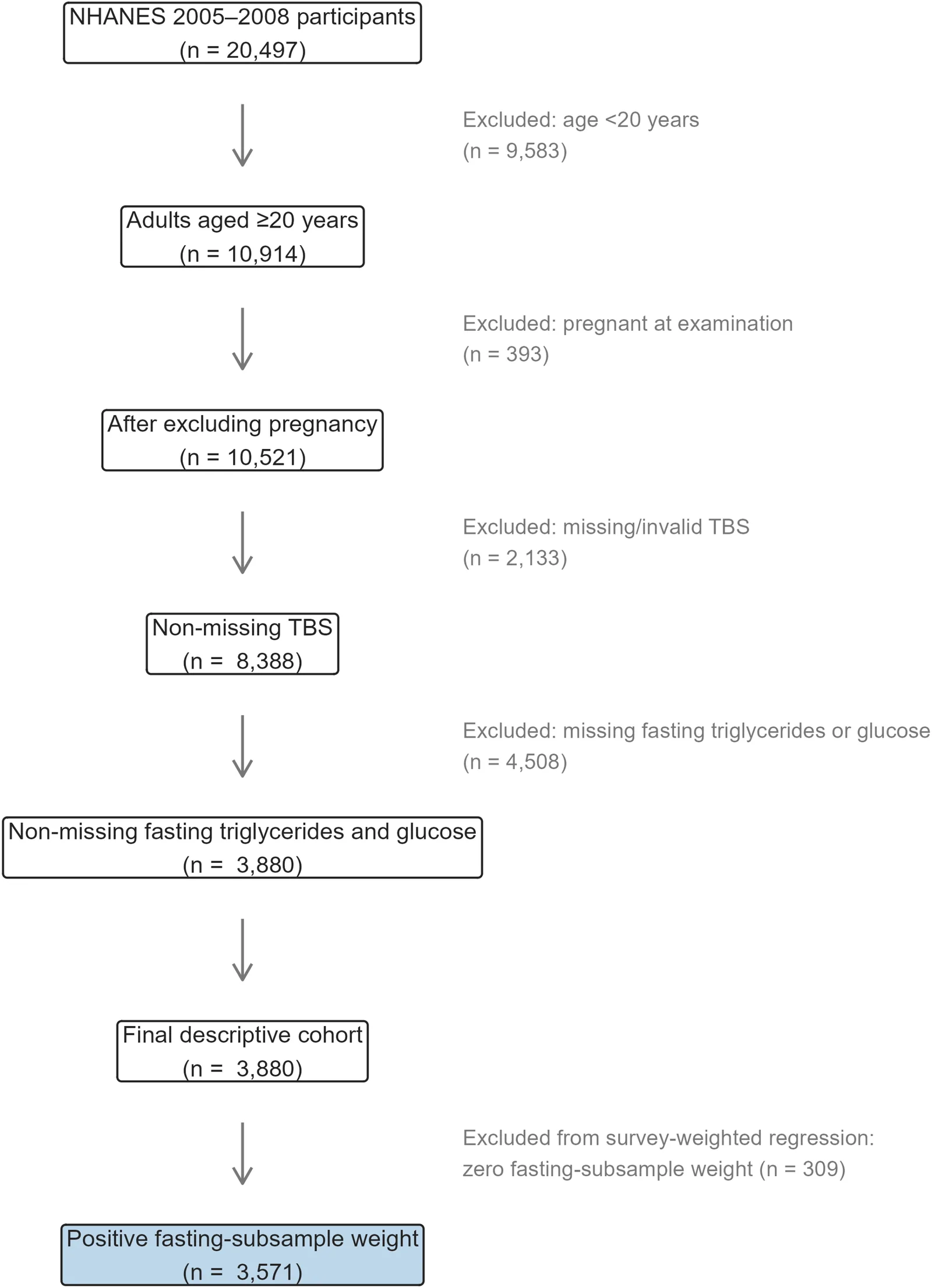
Selection of the NHANES 2005 to 2008 descriptive cohort and survey-weighted regression sample. The descriptive cohort included 3880 participants; 3571 with positive fasting-subsample weights contributed to survey-weighted regression. NHANES = National Health and Nutrition Examination Survey, TBS = trabecular bone score, TyG = triglyceride-glucose index.

### 2.2. Evaluation of the TyG index

The TyG index was calculated as ln(fasting triglycerides [mg/dL] × fasting glucose [mg/dL]/2).^[15]^ Although its component measurements are expressed in mg/dL, the logarithmic TyG index itself is dimensionless. TyG was analyzed continuously and, for descriptive purposes only, by quartiles.

### 2.3. Outcome variable

Total TBS, which was determined by examining grayscale variations in lumbar spine DXA scans, served as the study’s dependent variable. A Hologic QDR-4500A fan beam densitometer was used to obtain lumbar spine images. Then, for those who were 20 years of age and above, total TBS scores were computed using TBS software (Med-Imap SA TBS Calculator version 2.1.0.2; Medimaps Group SA).

### 2.4. Covariate

Prespecified covariates were age, sex, race/ethnicity, education, marital status, income-to-poverty ratio, body mass index, alanine aminotransferase (ALT), aspartate aminotransferase (AST), total cholesterol (TC), serum calcium, alcohol use, hypertension, diabetes, and smoking. In the primary analysis, diabetes was defined by self-reported physician diagnosis (DIQ010). In an exploratory laboratory-based glycemic-status classification, diabetes was defined as self-report or fasting glucose ≥126 mg/dL, prediabetes as fasting glucose 100 to 125 mg/dL without diabetes, and normoglycemia as fasting glucose <100 mg/dL without diabetes; this classification was used for the prespecified subgroup interaction analysis. Because BMD may lie on the pathway linking metabolic dysfunction to bone microarchitecture, femur and spine BMD were excluded from the primary model and included only in a sensitivity model. Serum creatinine was added in a separate renal-function sensitivity analysis.

### 2.5. Statistical analysis

Analyses incorporated the NHANES strata (SDMVSTRA), primary sampling units (SDMVPSU), and 4-year fasting-subsample weights (WTSAF2YR/2). Participants with zero fasting-subsample weight were retained in the descriptive cohort but excluded from survey-weighted regression. Covariate missingness was handled by chained-equation multiple imputation using 10 datasets (Table S2, Supplemental Digital Content 2); TBS, TyG, design variables, and weights were not imputed. The primary survey-weighted linear model adjusted for demographic, socioeconomic, clinical, and behavioral covariates but not BMD. Nonlinearity was evaluated using a restricted cubic spline with knots at the 5th, 35th, 65th, and 95th percentiles of TyG; the median TyG (8.66) was the reference. Wald tests assessed the overall TyG association and the nonlinear spline terms. A separate two-segment weighted linear model estimated a data-driven knot, which was treated as exploratory because no bootstrap or external validation was performed. Sensitivity analyses additionally adjusted for femur and spine BMD, added serum creatinine, and used complete cases. Collinearity was assessed using variance inflation factors (VIFs); weighted residual and influence diagnostics were also examined. Two-sided *P* < .05 was considered statistically significant. Analyses and figures were produced in R 4.5.2 (The R Foundation for Statistical Computing).

## 3. Results

### 3.1. Study population features

The descriptive cohort comprised 3880 adults; 3571 had positive fasting-subsample weights and contributed to survey-weighted models. The reconstructed source files showed missingness for education (0.05%), income-to-poverty ratio (6.57%), ALT and AST (0.93% each), total cholesterol (0.41%), calcium (0.39%), alcohol use (3.25%), hypertension and diabetes (0.13% each), smoking (0.10%), femur BMD (6.70%), and spine BMD (13.09%). Across covariates, missingness ranged from 0% to 13.09%; variables with missing values were multiply imputed in 10 datasets (Table S2, Supplemental Digital Content 2). Coefficient-level VIFs did not indicate severe multicollinearity (maximum VIF, 4.60 for ALT; 4.28 for AST; TyG VIF, 1.58); weighted residual and influence diagnostics were reviewed. Weighted characteristics by TyG quartile are shown in Table 1, and included-versus-excluded comparisons are shown in Table S1, Supplemental Digital Content 1.

**Table 1:** Weighted sample characteristics for the study.

	Q1 6.65–8.25	Q2 8.25–8.66	Q3 8.66–9.10	Q4 9.10–12.33	*P* value
Age (%)					<.001
<60	79.485	66.151	60.247	58.247	
≥60	20.515	33.849	39.753	41.753	
Gender (%)					<.001
Male	39.914	48.667	53.679	60.290	
Female	60.086	51.333	46.321	39.710	
Race (%)					<.001
Mexican American	5.281	8.169	8.552	10.178	
Other Hispanic	3.322	3.320	4.872	5.185	
Non-Hispanic White	68.724	72.945	72.296	72.484	
Non-Hispanic Black	17.150	10.652	7.646	5.932	
Other race	4.983	4.914	6.634	6.222	
Education level (%)					<.001
Less than high school	14.548	16.706	18.553	22.691	
High school	19.900	27.327	25.495	27.662	
More than high school	65.552	55.967	55.952	49.646	
Marital status (%)					.0013
Married or with partner	63.159	64.834	66.464	71.334	
Single	36.841	35.166	33.536	28.666	
PIR (%)					.2666
<1	10.659	9.836	10.220	11.923	
1–5	64.943	61.943	61.689	63.005	
≥5	24.398	28.221	28.091	25.072	
BMI (%)					<.001
Normal	52.165	34.572	24.408	13.093	
Overweight	28.351	36.842	37.899	37.938	
Obesity	19.485	28.856	37.693	48.969	
Alcohol status (%)					.084
Yes	72.908	75.819	74.586	70.887	
No	27.092	24.181	25.414	29.113	
Hypertension (%)					<.001
Yes	16.026	29.452	34.981	46.651	
No	83.974	70.548	65.019	53.349	
Diabetes (%)					<.001
Yes	1.614	3.816	6.753	20.612	
No	98.386	96.184	93.247	79.388	
Smoke (%)					<.001
Yes	41.117	49.162	54.473	55.356	
No	58.883	50.838	45.527	44.644	
Total femur BMD (g/cm^2^)	0.964 ± 0.147	0.970 ± 0.147	0.979 ± 0.151	1.008 ± 0.145	<.001
Total spine BMD (g/cm^2^)	1.038 ± 0.130	1.031 ± 0.130	1.034 ± 0.133	1.045 ± 0.139	.104
Total TBS	1.446 ± 0.115	1.402 ± 0.132	1.365 ± 0.142	1.310 ± 0.153	<.001
Total cholesterol (mg/dL)	181.169 ± 35.268	194.543 ± 34.677	203.855 ± 40.849	212.080 ± 46.390	<.001
Total calcium (mg/dL)	9.395 ± 0.335	9.399 ± 0.352	9.397 ± 0.337	9.456 ± 0.335	<.001
ALT (U/L)	21.909 ± 22.809	24.212 ± 15.759	27.129 ± 17.515	30.769 ± 18.999	<.001
AST (U/L)	24.449 ± 23.825	24.743 ± 11.240	25.741 ± 13.223	27.226 ± 11.734	<.001

For variables that fluctuate continuously, mean ± SD: the *P* value was calculated using a weighted linear regression model. % is a category variable. The *P*-value was ascertained by applying the weighted chi-square test.

ALT = alanine aminotransferase, AST = aspartate aminotransferase, BMD = bone mineral density, BMI = body mass index, TBS = trabecular bone score.

### 3.2. Correlation between TyG index and total TBS

In the multiple-imputation primary model without BMD, each 1-unit increase in TyG was associated with a 0.0097-unit lower TBS (95% confidence interval = −0.0182 to −0.0012; *P* = .026). The restricted cubic spline supported an overall nonlinear association (*P*-overall < .001; *P*-nonlinear < .001) relative to the median TyG value of 8.66 (Fig. 2). The association remained negative in the complete-case, serum-creatinine, and BMD-adjusted sensitivity models (Table 2). In the separate exploratory two-segment model, the estimated exploratory knot was 8.58 and requires external validation; slopes and sample sizes on both sides are reported in Table 3. The spline describes the continuous shape of the association, whereas the unvalidated two-segment knot is presented only as a descriptive analysis.

**Table 2:** Relationship between the trabecular bone score and the triglyceride-glucose index.

Model	β	95% CI	*P* value	Interpretation
Sensitivity: complete cases, no BMD	−0.0103	−0.0200 to −0.0007	.038	TBS difference per 1-unit increase in dimensionless TyG
Primary: 10 imputations, no BMD	−0.0097	−0.0182 to −0.0012	.026	TBS difference per 1-unit increase in dimensionless TyG
Sensitivity: 10 imputations, +femur/spine BMD	−0.0132	−0.0202 to −0.0062	<.001	TBS difference per 1-unit increase in dimensionless TyG
Sensitivity: 10 imputations, +creatinine	−0.0096	−0.0181 to −0.0011	.027	TBS difference per 1-unit increase in dimensionless TyG

The primary model used 10 multiply imputed datasets and adjusted for age, sex, race/ethnicity, education, marital status, income-to-poverty ratio, BMI, ALT, AST, total cholesterol, serum calcium, alcohol use, hypertension, diabetes, and smoking. BMD was omitted to reduce potential overadjustment. Sensitivity models added femur and spine BMD, used complete cases, or added serum creatinine. Analyses incorporated WTSAF2YR/2, SDMVSTRA, and SDMVPSU.

ALT = alanine aminotransferase, AST = aspartate aminotransferase, BMD = bone mineral density, BMI = body mass index, TBS = trabecular bone score, TyG = triglyceride-glucose index.

**Table 3:** Exploratory two-segment analysis of the association between triglyceride-glucose index and trabecular bone score.

Segment	n	β per 1-unit TyG	95% CI
TyG < 8.58	1599	0.0169	0.0043 to 0.0295
TyG ≥ 8.58	1972	−0.0251	−0.0339 to −0.0162

The exploratory two-segment model used the primary covariate set without BMD. The estimated exploratory knot at TyG 8.58 requires external validation because it was selected from the same dataset and was not bootstrap or externally validated; it is not a clinical threshold. TyG was analyzed as a continuous, dimensionless index.

BMD = bone mineral density, CI = confidence interval, TyG = triglyceride-glucose index.

**Figure 2.**
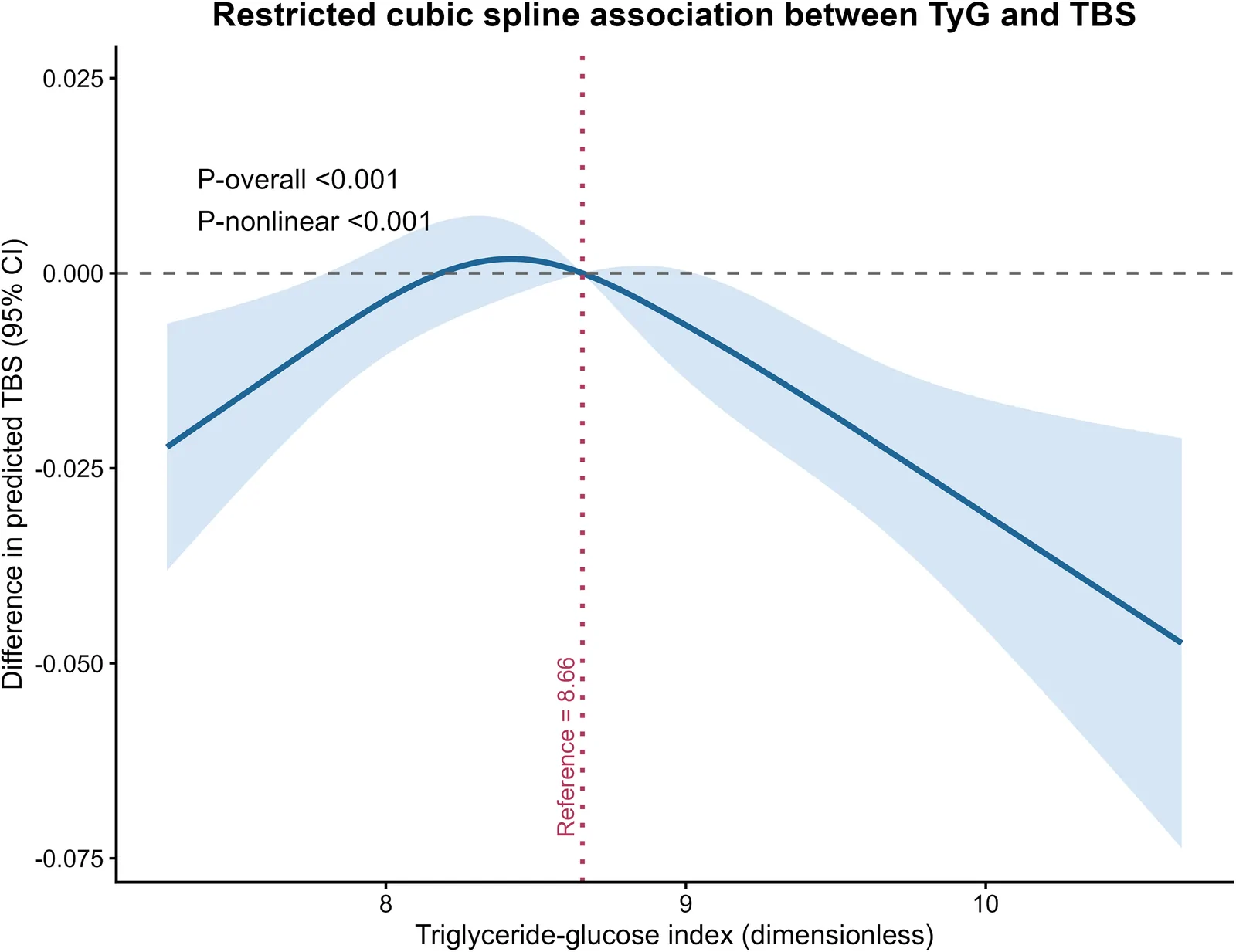
Survey-weighted restricted cubic spline association between the dimensionless TyG index and TBS. The blue line shows the difference in predicted TBS relative to the median TyG value (8.66), and shading shows the 95% confidence interval. The horizontal dashed line indicates no difference from the reference; the vertical dotted line marks the reference value. Knots were placed at the 5th, 35th, 65th, and 95th TyG percentiles. *P*-overall and *P*-nonlinear were obtained from pooled Wald tests across 10 imputed datasets. The primary covariate set excluded BMD. BMD = bone mineral density, TBS = trabecular bone score, TyG = triglyceride-glucose index.

### 3.3. Subgroup analysis

Exploratory survey-weighted subgroup estimates and exact unweighted subgroup sizes are shown in Figure 3. The inverse association was more pronounced in men than women (interaction *P* = .003) and in participants with obesity than those without obesity (interaction *P* < .001). Glycemic-status interaction evidence was weaker (*P* = .044), and interactions by age, hypertension, and alcohol use were not statistically significant. Given multiple exploratory comparisons and the cross-sectional design, these findings should be interpreted as hypothesis-generating.

**Figure 3.**
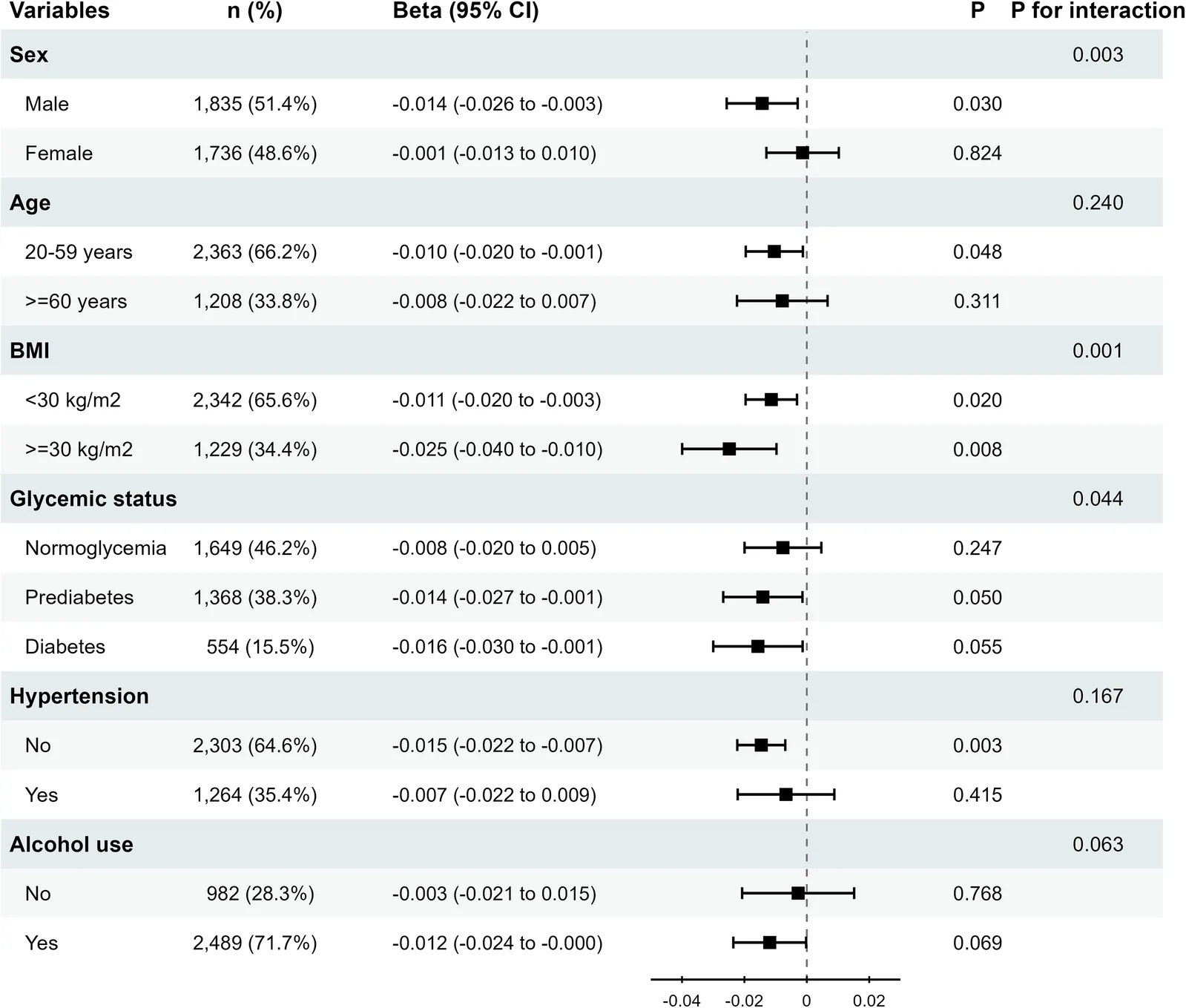
Survey-weighted subgroup associations between TyG and TBS. The table-forest layout reports unweighted n (percentage within each displayed subgroup variable), beta estimates per 1-unit increase in dimensionless TyG, 95% confidence intervals, subgroup-specific *P* values, and *P* values for interaction. Subgroup analyses were exploratory and used the primary covariate set without BMD. TyG was analyzed as a continuous, dimensionless index. BMD = bone mineral density, BMI = body mass index, TBS = trabecular bone score, TyG = triglyceride-glucose index.

## 4. Discussion

Using nationally representative NHANES 2005 to 2008 data, we found a modest inverse association between the dimensionless TyG index and TBS after incorporating fasting-subsample weights, strata, PSUs, and multiple imputation. Restricted cubic spline analysis indicated a nonlinear association (*P*-overall < 001; *P*-nonlinear < .001). The association persisted when BMD was omitted from the primary model and in sensitivity analyses with BMD, complete cases, and serum creatinine. These results support an association signal but do not establish temporal or causal effects.

Osteoporosis-related fractures are becoming more common in men over 65 and women over 55. These fractures lead to a number of bone-related problems and place a heavy financial burden on both individuals and society.^[16]^ It is crucial to remember that BMD by itself does not offer a complete evaluation of fracture risk because the skeleton’s capacity to tolerate stress and avoid fractures is also greatly influenced by the mechanical characteristics of bone tissue, such as its bone structure characteristics. Moreover, DXA measurements provide only a limited understanding of bone quality and are incapable of differentiating between trabecular and cortical bone.^[17,18]^ Conversely, TBS, the established methodology for evaluating skeletal microstructure observed in 2D DXA images,^[19]^ offers the ability to identify individuals with compromised microstructure despite having normal BMD. This approach is poised to become a valuable clinical tool for diagnosing osteoporosis and evaluating fracture risk,^[20]^ particularly in individuals with normal or high BMD. This includes patients with conditions like T2DM and obesity.^[21,22]^ The combination with TBS can thus improve the accuracy of fracture risk prediction.^[23,24]^

Several previous studies have independently highlighted the predictive role of TBS in assessing fracture risk, revealing a negative association between TBS and TC and glucose levels.^[8,25]^ Mitchell et al reported the connection between elevated fasting glucose and a smaller hip bone area, with the impact on BMD being less certain.^[26]^ In examining the relationship between the TyG index and diabetes, previous studies consistently exhibited a positive connection between the TyG index and diabetes.^[27]^ The TyG index, derived from fasting plasma glucose and TG, is indicative of glucose-lipid metabolism^[28]^ and holds significant relevance as a cardiovascular disease biomarker.^[29]^ Elevated levels of TG and TC have been positively connected to escalating osteoporosis risk, according to several studies.^[30]^ In the KNHANES 2008 to 2011 dataset, the TyG index, which is a composite reflection of glucose and TG, exhibited a negative association with femoral neck BMD in men ≥50 years who were not diabetic, as well as in postmenopausal women.^[31]^ The TyG index and BMD have been the subject of numerous recent studies,^[32]^ however, the clinical evidence linking the TyG index and TBS within the general population is still sparse and unconvincing. In order to determine the TyG index’s predictive usefulness for osteoporosis and fracture risk, our study will therefore look into the correlation between the TyG index and TBS.

TyG index may lead to bone microstructural disruption in patients through a variety of mechanisms, resulting in decreased bone turnover. Hyperglycemia disrupts the delicate balance between bone formation and resorption, inhibits osteoblast differentiation, suppresses osteocalcin and gene expressions involved in osteoclast maturation, and slows down bone turnover, all of which increase the risk of fractures. Furthermore, high blood sugar causes elevation of advanced glycation end products (AGEs), which interact with the receptor for advanced glycation end products on osteoblasts and attach to collagen in bone. Through the nuclear factor κB pathway, this interaction causes the production of inflammatory and oxidative mediators, which in turn compromises bone’s biomechanical qualities, lowers bone mass, and increases the risk of fractures.^[33,34]^ Furthermore, it decreases the production of type I collagen, encourages the development of weak connections between collagen fibers, and inhibits the expression, differentiation, and mineralization of osteoblast phenotypes. When all of these factors are taken into account, bone resorption mediated by osteoclasts increases and bone strength decreases.^[35,36]^ Studies have shown that altered insulin-like growth factor 1 (IGF-1) levels are also associated with skeletal abnormalities.^[37]^ In addition, when glucose and AGE levels are elevated, they lead to a decrease in IGF-1 activity, indicating that glucose and AGE levels can affect bone through the action of IGF-1.^[38,39]^ Moreover, changes in the local microenvironment caused by glucose and lipid metabolism lead to increased levels of proinflammatory cytokines such as tumor necrosis factor and interleukin 6, which in turn activate osteoclast formation and inhibit osteoblast differentiation, and also lead to osteoporosis.^[40]^

Recent studies have also reported associations between TyG-related metabolic indices and BMD or fracture outcomes in other populations,^[41]^ whereas research on lipid biomarkers supports the broader relationship between lipid metabolism and osteoporosis.^[42]^ Alterations in BMD and TBS among individuals with diabetes have likewise been documented.^[43]^

Restricted cubic spline analysis showed that the adjusted TyG-TBS association was nonlinear, with the curve centered at the median TyG value of 8.66. A separate two-segment analysis estimated an exploratory knot at 8.58, which requires external validation. However, the knot was selected from the same cross-sectional dataset and was not externally or bootstrap validated. It should therefore be viewed as an exploratory description rather than a treatment target. The present data cannot determine whether lowering TGs or glucose would improve bone microarchitecture or reduce fracture risk. A particularly striking finding in our data is the discordance between BMD and TBS across increasing TyG levels. While total spine BMD showed no significant change across TyG quartiles (*P* = .104), TBS exhibited a graded and substantial decline (from 1.446 to 1.310, *P* < .001). This discordance has 2 important implications. First, it suggests that insulin resistance and lipotoxicity, reflected by TyG, may preferentially affect trabecular microarchitecture through non-mineral pathways (e.g., advanced glycation end-products, oxidative stress, and marrow adiposity) rather than altering bulk mineral density. Second, from a clinical standpoint, relying solely on BMD in metabolically unhealthy patients may provide a falsely reassuring picture. Our findings reinforce that TBS should be particularly considered in obese, hypertensive, or dyslipidemic patients, as TyG elevation identifies those with “normal BMD but poor bone quality” – a subgroup at potentially under-recognized fracture risk.

Strengths include a nationally representative sample, standardized NHANES measurements, use of TBS as a bone-quality outcome, correct incorporation of fasting-subsample weights, strata and PSUs, and sensitivity analyses addressing missing data, potential BMD overadjustment, renal function, and model diagnostics. Several limitations remain. First, the cross-sectional design precludes temporal or causal inference. Second, exclusion related to missing TBS and fasting laboratory measurements may cause selection bias; included-versus-excluded comparisons are provided, but residual selection cannot be ruled out. Third, the exploratory knot was not bootstrap or externally validated and should not be interpreted as a clinical threshold. Fourth, despite extensive covariate adjustment, we lacked data on bone-active medications (e.g., bisphosphonates, glucocorticoids, and hormone replacement therapy), menopausal status, vitamin D, and detailed physical activity. If these unmeasured factors are associated with both higher TyG and lower TBS, they could overestimate the association. Conversely, some factors may act as mediators rather than confounders, in which case direct adjustment would be inappropriate. Residual confounding therefore remains an important threat and could bias the association in either direction. Fifth, subgroup analyses were exploratory and not adjusted for multiplicity. Finally, TBS and TyG were assessed at one examination, and the 2005 to 2008 cycles may not reflect contemporary exposure distributions.

From a clinical practice standpoint, the TyG index possesses distinct advantages over sophisticated biomarkers: it is calculated instantaneously from existing laboratory reports without additional cost or patient inconvenience. Thus, even a modest and exploratory association, as observed here, could hold clinical value if it channels higher-risk patients toward targeted DXA/TBS assessment and thereby rationalizes the use of limited DXA/TBS resources.

## 5. Conclusion

Higher TyG was associated with lower TBS, with stable BMD but declining TBS across TyG quartiles – suggesting TyG may signal bone quality deterioration rather than mineral loss. The exploratory threshold of 8.58 requires validation. TyG, obtained from routine labs at no extra cost, could prompt TBS evaluation in metabolically high-risk patients, especially obese men, even when BMD is normal. A cross-sectional design precludes causality; prospective studies are needed to test TyG’s value in fracture risk stratification.

## Acknowledgments

The authors acknowledge the work of the NCHS in producing the data for the NHANES.

## Author contributions

**Conceptualization:** Haichao Xu, Hao Xu, Jianyang Luo, Jianfeng Zhao.

**Data curation:** Haichao Xu, Hao Xu, Jianyang Luo.

**Formal analysis:** Haichao Xu, Hao Xu, Jianyang Luo, Jianfeng Zhao.

**Funding acquisition:** Haichao Xu, Hao Xu, Jianyang Luo, Jianfeng Zhao.

**Investigation:** Haichao Xu, Hao Xu, Jianyang Luo.

**Methodology:** Haichao Xu, Hao Xu, Jianyang Luo, Jianfeng Zhao.

**Project administration:** Haichao Xu, Jianfeng Zhao.

**Resources:** Haichao Xu, Jianfeng Zhao.

**Software:** Haichao Xu, Jianfeng Zhao.

**Supervision:** Haichao Xu, Jianfeng Zhao.

**Validation:** Haichao Xu, Jianfeng Zhao.

**Visualization:** Haichao Xu, Jianfeng Zhao.

**Writing – original draft:** Haichao Xu, Jianfeng Zhao.

**Writing – review & editing:** Haichao Xu, Jianfeng Zhao.




