# Supplementary material for: Association between triglyceride-glucose index and total trabecular bone score in US adults: A cross-sectional study
Source: Medicine (Baltimore). 2026 Oct 23;105(43):e51014. doi: 10.1097/MD.0000000000051014 (PMC13706339; doi:10.1097/MD.0000000000051014)
Supplement: Supplementary file 1 [file medi-105-e51014-s001.docx]

Supplementary Table S1

Included versus excluded age-eligible, nonpregnant adults

| **Characteristic** | **Included** | **Excluded** | **Available n (included/excluded)** |
| --- | --- | --- | --- |
| Age, years | 46.84 (0.54) | 47.05 (0.43) | 3880/6641 |
| BMI, kg/m² | 28.05 (0.12) | 28.87 (0.18) | 3880/6026 |
| TyG, dimensionless | 8.68 (0.02) | 8.79 (0.02) | 3880/682 |
| TBS | 1.38 (0.00) | 1.38 (0.00) | 3880/4508 |
| Total femur BMD, g/cm² | 0.98 (0.00) | 0.97 (0.00) | 3620/4600 |
| Total spine BMD, g/cm² | 1.04 (0.00) | 1.04 (0.00) | 3372/3917 |
| Male, % | 50.3 | 48.0 | 3880/6641 |
| Non-Hispanic White, % | 71.6 | 70.5 | 3880/6641 |
| Diabetes, % | 7.9 | 8.5 | 3875/6634 |
| Hypertension, % | 31.3 | 30.0 | 3875/6625 |

*Values are survey-weighted mean (SE) or weighted percentage. Comparison is restricted to adults aged ≥20 years who were not pregnant at examination. Variable-specific available n values are shown because exclusion was driven by missing TBS and fasting laboratory data.*
