# Supplementary material for: Association between triglyceride-glucose index and total trabecular bone score in US adults: A cross-sectional study
Source: Medicine (Baltimore). 2026 Oct 23;105(43):e51014. doi: 10.1097/MD.0000000000051014 (PMC13706339; doi:10.1097/MD.0000000000051014)
Supplement: Supplementary file 2 [file medi-105-e51014-s002.docx]

Supplementary Table S2

Missing covariate data in the 3,880-person descriptive cohort

| **Variable** | **Missing n** | **Missing %** |
| --- | --- | --- |
| TBS | 0 | 0.00 |
| TyG | 0 | 0.00 |
| age | 0 | 0.00 |
| sex | 0 | 0.00 |
| race | 0 | 0.00 |
| education | 2 | 0.05 |
| married | 0 | 0.00 |
| pir | 255 | 6.57 |
| bmi | 0 | 0.00 |
| alt | 36 | 0.93 |
| ast | 36 | 0.93 |
| cholesterol | 16 | 0.41 |
| calcium | 15 | 0.39 |
| creatinine | 16 | 0.41 |
| alcohol | 126 | 3.25 |
| hypertension | 5 | 0.13 |
| diabetes | 5 | 0.13 |
| smoking | 4 | 0.10 |
| femur_bmd | 260 | 6.70 |
| spine_bmd | 508 | 13.09 |

*Covariates were imputed by chained equations in 10 datasets. TBS, TyG, weights, strata, and PSUs were not imputed.*
